# A multi‐institutional analysis of a general pelvis continuous Hounsfield unit synthetic CT software for radiotherapy

**DOI:** 10.1002/acm2.13205

**Published:** 2021-02-22

**Authors:** Victoria Y. Yu, Jani Keyrilainen, Sami Suilamo, Ilyes Beslimane, Alex Dresner, Aleksi Halkola, Uulke A. Van der Heide, Neelam Tyagi

**Affiliations:** ^1^ Memorial Sloan Kettering Cancer Center New York NY USA; ^2^ Department of Oncology and Radiotherapy & Department of Medical Physics Turku University Hospital Turku Finland; ^3^ Philips Healthcare Eindhoven The Netherlands; ^4^ Department of Radiation Oncology The Netherlands Cancer Institute Amsterdam The Netherlands

**Keywords:** mDixon, MR‐only, multi‐institutional, pelvis, radiotherapy, synthetic CT

## Abstract

**Purpose:**

To validate a synthetic computed tomography (sCT) software with continuous HUs and large field‐of‐view (FOV) coverage for magnetic resonance imaging (MRI)‐only workflow of general pelvis anatomy in radiotherapy (RT).

**Methods:**

An sCT software for general pelvis anatomy (prostate, rectum, and female pelvis) has been developed by Philips Healthcare and includes continuous HUs assignment along with large FOV coverage. General pelvis sCTs were generated using a two‐stack T1‐weighted mDixon fast‐field echo (FFE) sequence with a superior‐inferior coverage of 36 cm. Seventy‐seven prostate, 43 rectum, and 27 gynecological cases were scanned by three different institutions. mDixon image quality and sCTs were evaluated for soft tissue contrast by using a confidence level scale from 1 to 5 for bladder, prostate/rectum interface, mesorectum, and fiducial maker visibility. Dosimetric comparison was performed by recalculating the RT plans on the sCT after rigid registration. For 12 randomly selected cases, the mean absolute error (MAE) between sCT and CT was calculated to evaluate HU similarity, and the Pearson correlation coefficients (PCC) between the CT‐ and sCT‐generated digitally reconstructed radiographs (DRRs) were obtained for quantitative comparison. To examine geometric accuracy of sCT as a reference for cone beam CT (CBCT), the difference between bone‐based alignment of CBCT to CT and CBCT to sCT was obtained for 19 online‐acquired CBCTs from three patients.

**Results:**

Two‐stack mDixon scans with large FOV did not show any image inhomogeneity or fat‐water swap artifact. Fiducials, Foley catheter, and even rectal spacer were visible as dark signal on the sCT. Average visibility confidence level (average ± standard deviation) on the sCT was 5.0 ± 0.0, 4.6 ± 0.5, 3.8 ± 0.4, and 4.0 ± 1.1 for bladder, prostate/rectum interface, mesorectum and fiducial markers. Dosimetric accuracy showed on average < 1% difference with the CT‐based plans for target and normal structures. The MAE of bone and soft tissue between the sCT and CT are 120.9 ± 15.4 HU, 33.4 ± 4.1 HU, respectively. Average PCC of all evaluated DRR pairs was 0.975. The average offset between CT and sCT as reference was (LR, AP, SI) = (0.19 ± 0.35, 0.14 ± 0.60, 0.44 ± 0.54) mm.

**Conclusions:**

The continuous HU sCT software‐generated realistic sCTs and DRRs to enable MRI‐only planning for general pelvis anatomy.

## 1 INTRODUCTION

Magnetic resonance imaging (MRI) has been an integral part of the radiotherapy (RT) process for more than a decade due to its excellent soft tissue contrast. Multiple studies have shown the superiority of MRI for target and normal tissue segmentation in external beam RT by demonstrating reduced interobserver variability in contours compared with those obtained from computed tomography (CT).^1^, ^2^, ^3^ The current RT simulation process relies on target and organs‐at‐risk (OAR) segmentation on MRI followed by transfer of contours to CT via image registration. Although incorporating MRI decreases over‐segmentation of structures as compared with CT‐based segmentation, a combined CT‐MRI method is challenging due to errors introduced by mis‐registration of the image sets and the changes to the shape and location of the soft tissues, for example, bladder, rectum, and seminal vesicles that are inherent when acquiring multiple image sets.^4^, ^5^, ^6^ Because of the challenges in target delineation, registration uncertainties, and changes in anatomy due to temporal variations, a workflow in which MRI is the primary and sole imaging modality is highly preferable to a combined CT and MRI workflow.

An important component of MRI‐only workflow is generation of synthetic CTs (sCT). An sCT software for general pelvis anatomy (prostate, rectum, and female pelvis) has been developed by Philips Healthcare and includes continuous Hounsfield units (HU) assignment along with large field‐of‐view (FOV) coverage. The method is an extension of the earlier MRCAT (magnetic resonance for calculating attenuation) prostate software package that is currently implemented clinically at various institutions.^7^, ^8^, ^9^, ^10^ MRCAT prostate uses a single 3D mDIXON XD fast‐field echo (FFE) scan to generate sCTs. A constrained shape model is used to estimate body contour as well as segment bone structures. Bulk densities are assigned to five different tissue types (air, fat, soft tissue, spongy bone, and cortical bones). MRCAT is limited to a superior‐inferior extent of 30 cm and up to L4 vertebrae only. MRCAT prostate has recently been modified using a two‐stack mDIXON sequence and developed to generate sCTs using continuous HU generation as well as for general pelvis anatomy. For many high‐risk prostate, gynecological, and rectum cases nodal volumes are treated which can extent up to L1–L3. Scanning larger volume in superior‐inferior direction is challenging due to the concerns for geometrical distortions as well as scan homogeneity. The goal of this study was to assess the image quality, dosimetric and geometric accuracy of an sCT software with continuous HUs and large FOV coverage for MRI‐only workflow of a general pelvis anatomy.

## 2 MATERIALS AND METHODS

### 2.A Cases analyzed

Seventy‐seven prostate, 43 rectum, and 27 gynecological cases were scanned by three different institutions, namely Memorial Sloan Kettering Cancer Center (MSKCC, New York, NY, USA), Turku University Hospital (TUH, Turku, Finland) and the Netherlands Cancer Institute (NKI, Amsterdam, The Netherlands), on a 1.5T or 3T MRI scanner. Cases included both prone and supine simulation setup. Dose prescription varied from standard fractionation for prostate, rectum, and gynecological cases to hypofractionation for prostate and rectum. Institution‐specific scanner, patient, and treatment planning details of three institutions that participated in dosimetry and/or image quality evaluations are shown in Table 1.

**TABLE 1:** Institution specific patient details.

	MSKCC	TUH	NKI
Field strength	3 T	1.5 T	3T
Treatment planning system	Eclipse	Eclipse	Pinnacle
Dose prescription			
Prostate	800 cGy × 5	300 cGy × 20, 725 cGy × 5	220cGy × 35, 725 cGy × 5
Rectum	180/200 cGy × 25	180/190 cGy × 25/26, 500 cGy × 5	200 cGy × 25, 500 cGy × 5
Gynecological	180 cGy × 28	180/190 cGy × 25/26	200 cGy × 25
Tx machine	Varian	Varian	Elekta

MSKCC: Memorial Sloan‐Kettering Cancer Center; TUH: Turku University Hospital; NKI: Netherland Cancer Institute.

### 2.B Algorithm details

**FIG. 1:**
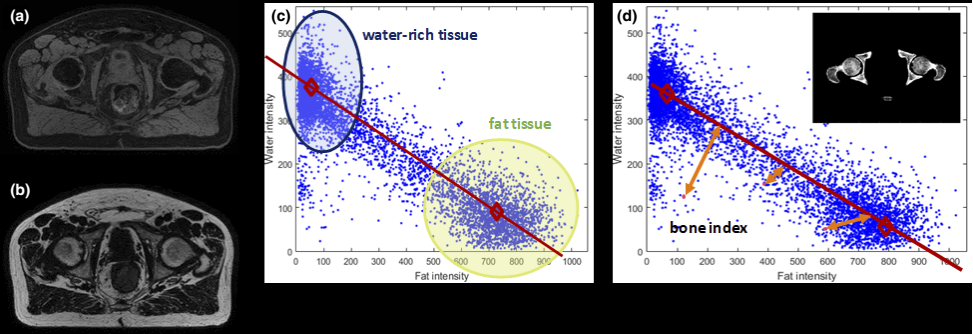
Intensity‐based classification using mDixon MRI to generate sCT. (a) water source image (b) fat source image. (c) Water intensity vs. fat intensity scatter plot illustrating the estimated centers of water‐rich and fat‐rich voxels for linear and continuous soft tissue voxel mapping. (d) Illustration of bone HU classification based on distance from water‐fat classification line (MRI: magnetic resonance imaging; sCT: synthetic computed tomography, HU: Hounsfield unit).

General pelvis sCTs were generated using a two‐stack T1‐weighted mDIXON FFE sequence (TR/TE1/TE2 = 4.7/1.4/2.8 ms, voxel size = 1.40 × 1.40 × 1.40 mm^3^, FOV = 368 × 552 × 360 mm^3^ bandwidth = 866.3 Hz) on 1.5T and 3T Philips Ingenia MR‐RT scanners with a superior‐inferior coverage of 36 cm. A two‐stack acquisition was employed to acquire large FOV in superior–inferior (SI) dimension and potentially remove any geometric distortion. MRCAT general pelvis for sCT generation includes both male and female bone model shape variations. Body outline and bones are segmented from mDIXON in‐phase and water images. The bones are identified using a model‐based segmentation. Soft tissue is classified as everything within the body outline that is outside of the segmented bones. A continuum of HUs is assigned separately in the bone and soft tissue compartments. Depending on the fat and water intensities of the voxels, these continua span the range from dense cortical bone to light spongy bone and fat to muscle tissue, respectively, guided by comparison to CT scans. Figure 1 shows further details on the intensity‐based classification in the mDIXON image for sCT generation. As a first step, centers of water‐rich and fat‐rich voxels on a water intensity vs fat intensity scatter plot are estimated, illustrated as red diamond markers in Fig. 1(c). The HU values for the soft tissue voxels are mapped continuously between water and fat clusters, as demonstrated by the red line in Fig. 1(c). Dense bone manifests on the mDIXON image as voxels with low signal intensity. Hence, the voxels within the bone mask are classified based on the distance of the voxels from the water‐fat classification line, as shown in Fig. 1(d).

### 2.C Data analysis

Image quality of the mDIXON source images and sCTs from general pelvis cases from all three institutions were evaluated for soft tissue contrast by using a confidence level scale from 1 to 5 for bladder, prostate/rectum interface, mesorectum, and fiducial maker visibility. Scoring specifications are as follows, with 1: very doubtful, 2: doubtful, 3: undetermined, 4: confident, 5: very confident. Scoring of all cases was performed by an expert anatomist.

To evaluate the accuracy of the sCT for patient treatment planning, the treatment plan and structure set from the original planning CT was transferred to the sCT after rigid registration with the planning CT. This analysis was performed with a subset of patients from MSKCC and TUH (n = 110). Dosimetric comparison was performed by recalculating the intensity‐modulated radiation therapy / volumetric‐modulated arc therapy (IMRT/VMAT) plans on the sCT. The following structures and dosimetric quantities were evaluated: planning target volume (PTV) (D_max_, D_mean_, D_95_), bladder (D_max_, D_mean_, D_53_, D_1cc_), rectum (D_max_, D_mean_, D_53_, D_1cc_), and femurs (D_max_). The percent difference relative to metrics corresponding to the primary CT was used.

To evaluate the HU agreement between sCT to CT, mean absolute error (MAE) for bone, soft tissue, and total body contour were calculated for 12 randomly selected patients from MSKCC and TUH. To establish voxel‐by‐voxel association between the sCT and CT, deformable image registration was performed in MIM Vista^TM^ (version 6.9.6, MIM Software Inc, Cleveland, OH, USA) software. The bony anatomy was manually contoured in MIM for each case. Soft tissue contour was defined as all tissue within the external body contour that is outside of the contoured bone. The registered scans and contours were transferred to MATLAB (version 2019b, The MathWorks Inc., Natick, MA, USA) software for all MAE calculations.

To further evaluate the difference between the widely utilized bulk density synthetic CT (sCT‐BD) from the new continuous HU approach, MAE to the CT for both methods were also performed for two cases in which sCT‐BD images were also acquired. Since sCT‐BD has a shorter superior‐inferior FOV of 30 cm, contours utilized in this comparison for MAE calculations were adjusted to ensure fair comparison.

To evaluate the accuracy of sCT as a reference for 2D image‐guided RT (IGRT), digitally reconstructed radiographs (DRRs) were generated from sCT in Varian Eclipse treatment planning system and qualitatively compared with CT‐generated DRRs. Quantitative analysis was performed using bony registration between DRRs generated from both the sCT and CT and calculating the Pearson correlation coefficient (PCC) metrics for all registered DRR pairs. PCC metric of DRRs in the anterior–posterior (AP) and right–left (RL) lateral projections for the same 12 cases utilized in MAE evaluations were evaluated. To examine geometric accuracy of sCT as a reference for cone beam CT (CBCT), the difference between bone‐based alignment of CBCT to CT and CBCT to sCT was obtained for 19 online‐acquired CBCTs from three patients.

## 3 RESULTS

**FIG. 2:**
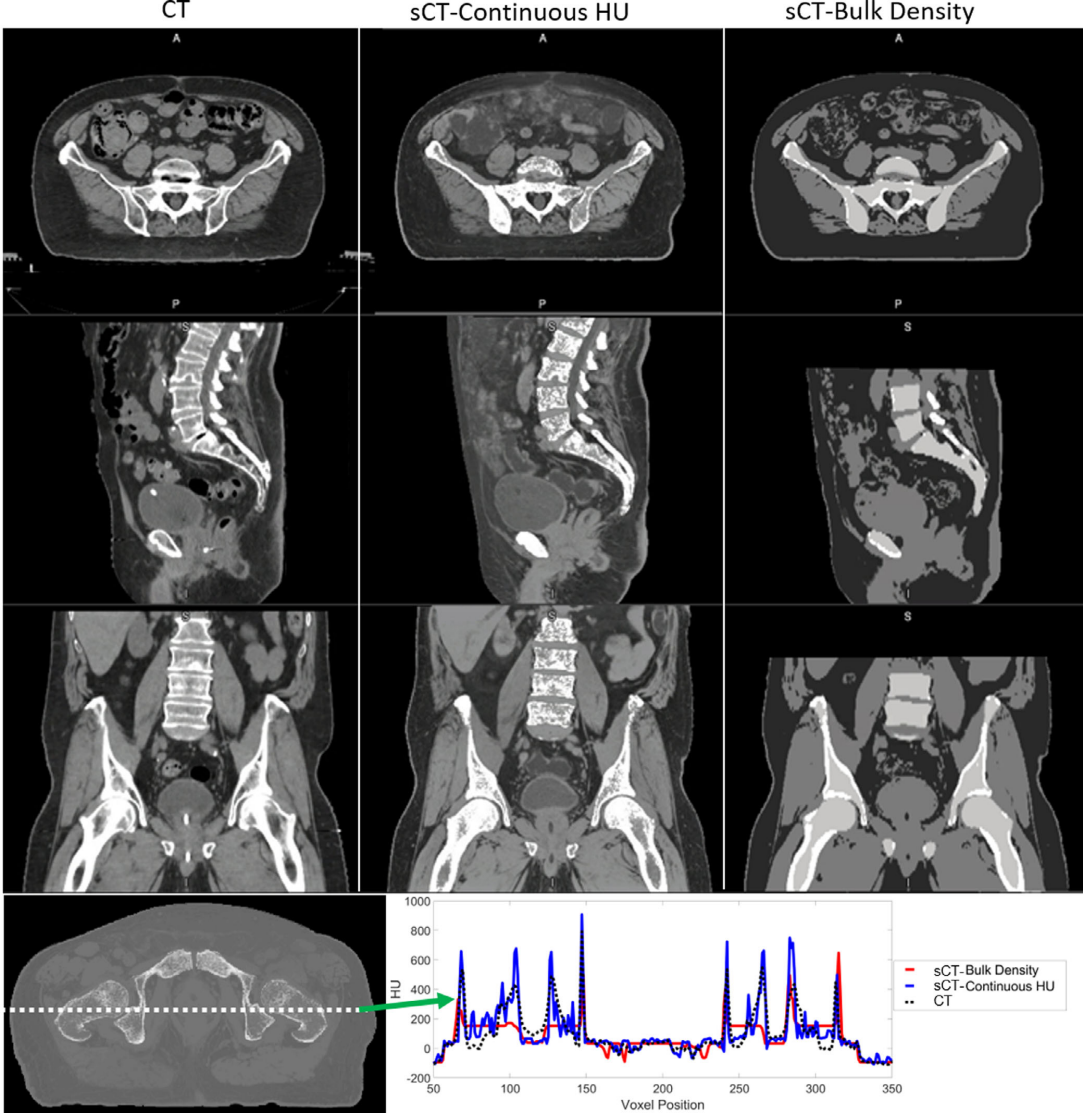
An example case comparing planning CT, continuous HU synthetic CT and the bulk density synthetic CT. HU profile comparison between CT and the continuous HU and bulk density synthetic CTs. CT: black dashed line, continuous HU synthetic CT: blue, bulk density synthetic CT: red (CT: computed tomography, HU: Hounsfield unit, A: anterior; P: posterior, S: superior, I: inferior).

Figure 2 shows the original CT, MRCAT general pelvis sCT‐cHU, and MRCAT prostate sCT‐BD, and an example HU profile comparing the three images from an example case. sCT‐cHU looks significantly more similar to the planning CT, and offers a longer FOV in the SI direction, when compared with the widely utilized sCT‐BD. Example HU profile of all three images overlaid also further demonstrates that sCT‐cHU more closely agrees with that of the CT.

### 3.A mDIXON MRI and synthetic CT image quality

**FIG. 3:**
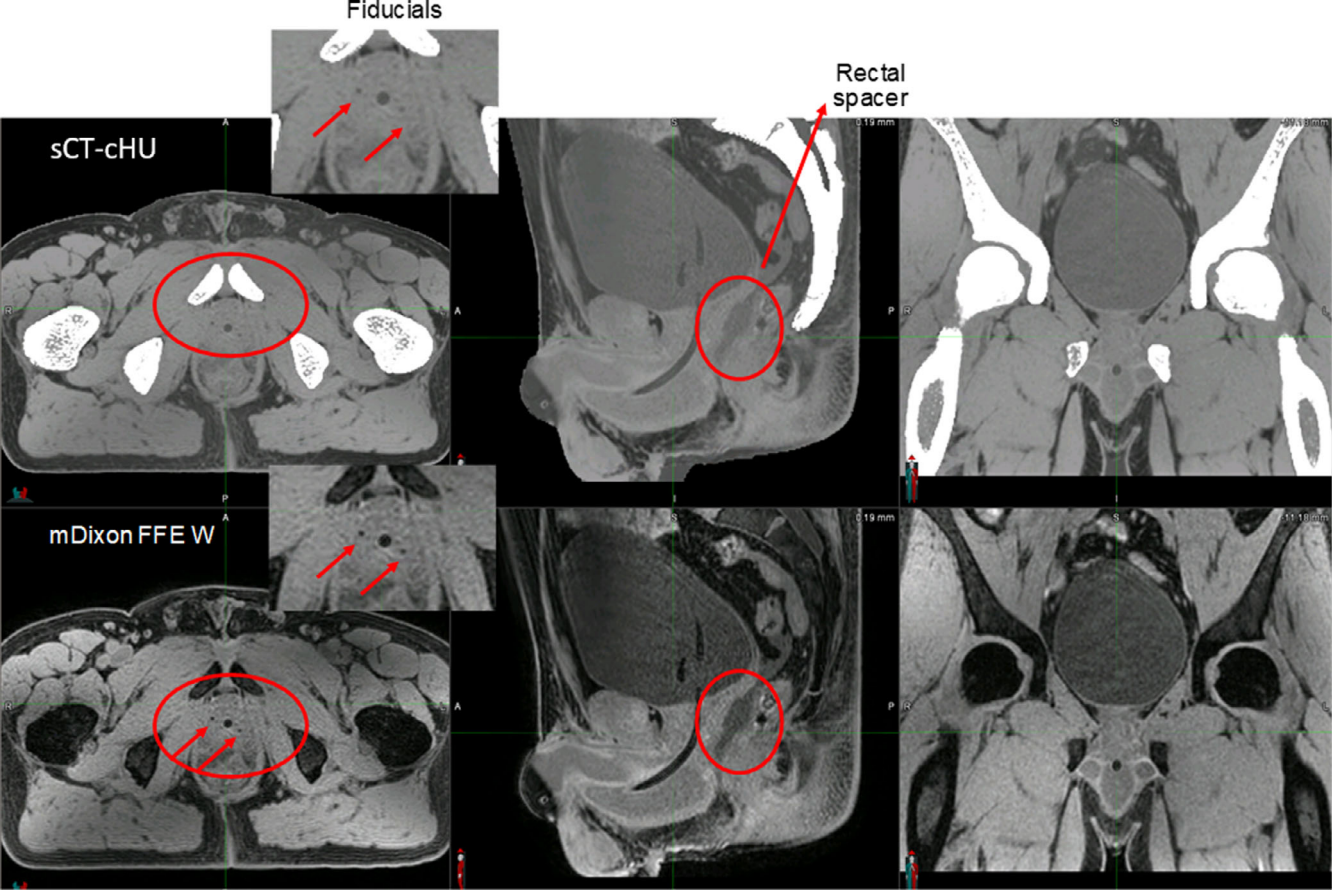
Soft tissue visibility on continuous HU synthetic CT as well as source MRI (mDIXON FFE Water). Images were acquired with 3T field strength (HU: Hounsfield unit, CT: computed tomography, MRI: magnetic resonance imaging, FFE: fast‐field echo, A: anterior, P: posterior, S: superior, I: inferior).

Large FOV acquisition is challenging in terms of geometric accuracy in the SI direction. Two‐stack mDixon acquisition mitigated that. Large FOV did not show any image inhomogeneity or fat‐water swap artifact based on visual inspection. Continuous HUs provided soft tissue and bone contrast on the sCT that is comparable to CT. Fiducials and Foley catheter were visible as dark signal on the sCT. Even rectal spacer showed a slightly darker contrast on sCT compared with nearby soft tissue as shown in Fig. 3. Spacer is typically not seen on the CT. sCTs reconstructed successfully in both prone and supine positions as well as a slightly frog‐legged position used for gynecological setups.

**FIG. 4:**
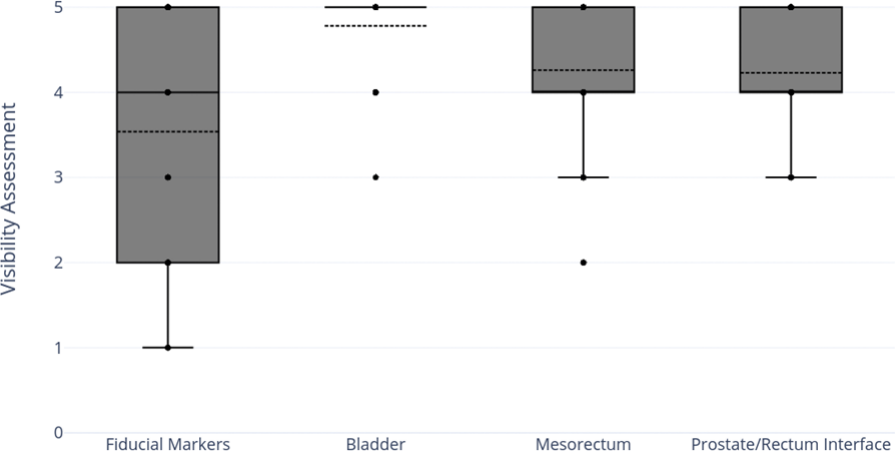
Average visibility confidence level score on the continuous HU synthetic CT for fiducial markers, bladder, mesorectum, and prostate/rectum interface (n = 77) (HU: Hounsfield unit, CT: computed tomography).

Figure 4 shows the boxplot for all confidence level scores for soft tissue visualization on synthetic CT images (n = 77). Average visibility confidence level on the sCT was 5.0 ± 0.0, 4.6 ± 0.5, 3.8 ± 0.4, and 4.0 ± 1.1 for bladder, prostate/rectum interface, mesorectum and fiducial markers. Bladder always performed well followed by prostate/rectum interface and mesorectum. Fiducials did not always show up as dark signal on the sCT. Mesorectum was in general difficult to visualize on gynecological cases. This could be a result of extensive disease outside the utero‐cervix region for the cases chosen in this evaluation.

### 3.B Dosimetric accuracy

**FIG. 5:**
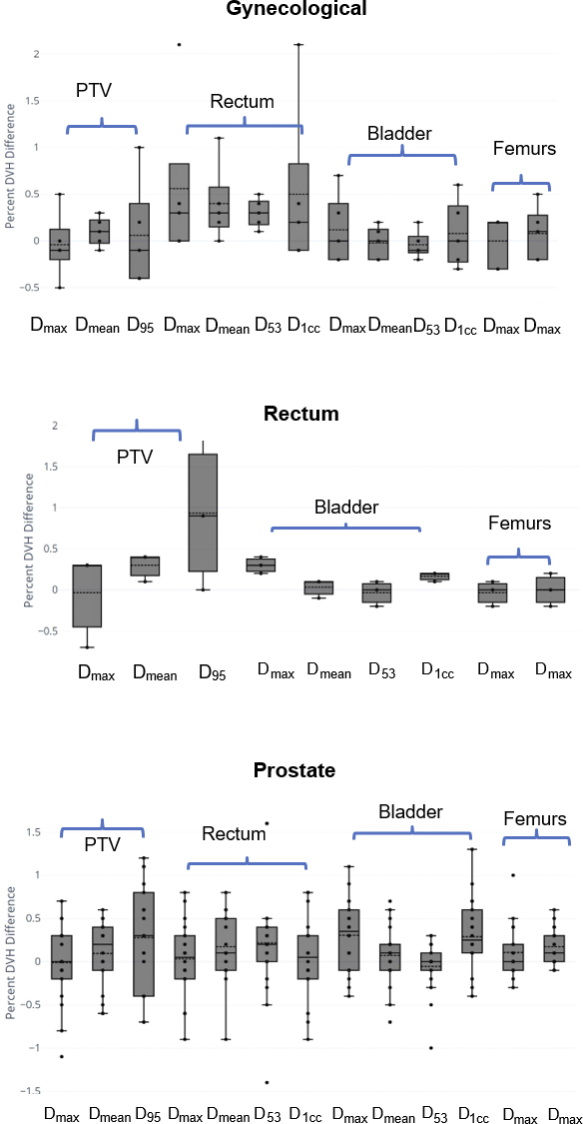
Boxplots showing dosimetric comparison between sCT and planning CT for gynecological, rectum, and prostate cases. Percent DVH difference = ($\frac{sCT‐CT}{\mathrm{CT}}$). Multiple dose metrics for PTV, bladder, rectum, and femurs were evaluated (n = 110) (PTV: planning target volume; DVH: dose‐volume histogram; sCT: synthetic computed tomography; CT: computed tomography).

Figure 5 shows the box plots of percent dose difference for various structures evaluated for different anatomical sites (n = 110). Overall, dosimetric accuracy showed on average < 1% difference with the CT‐based plans for target and normal structures.

### 3.C Synthetic CT: HU accuracy, DRR similarity, and CBCT bony alignment accuracy

**FIG. 6:**
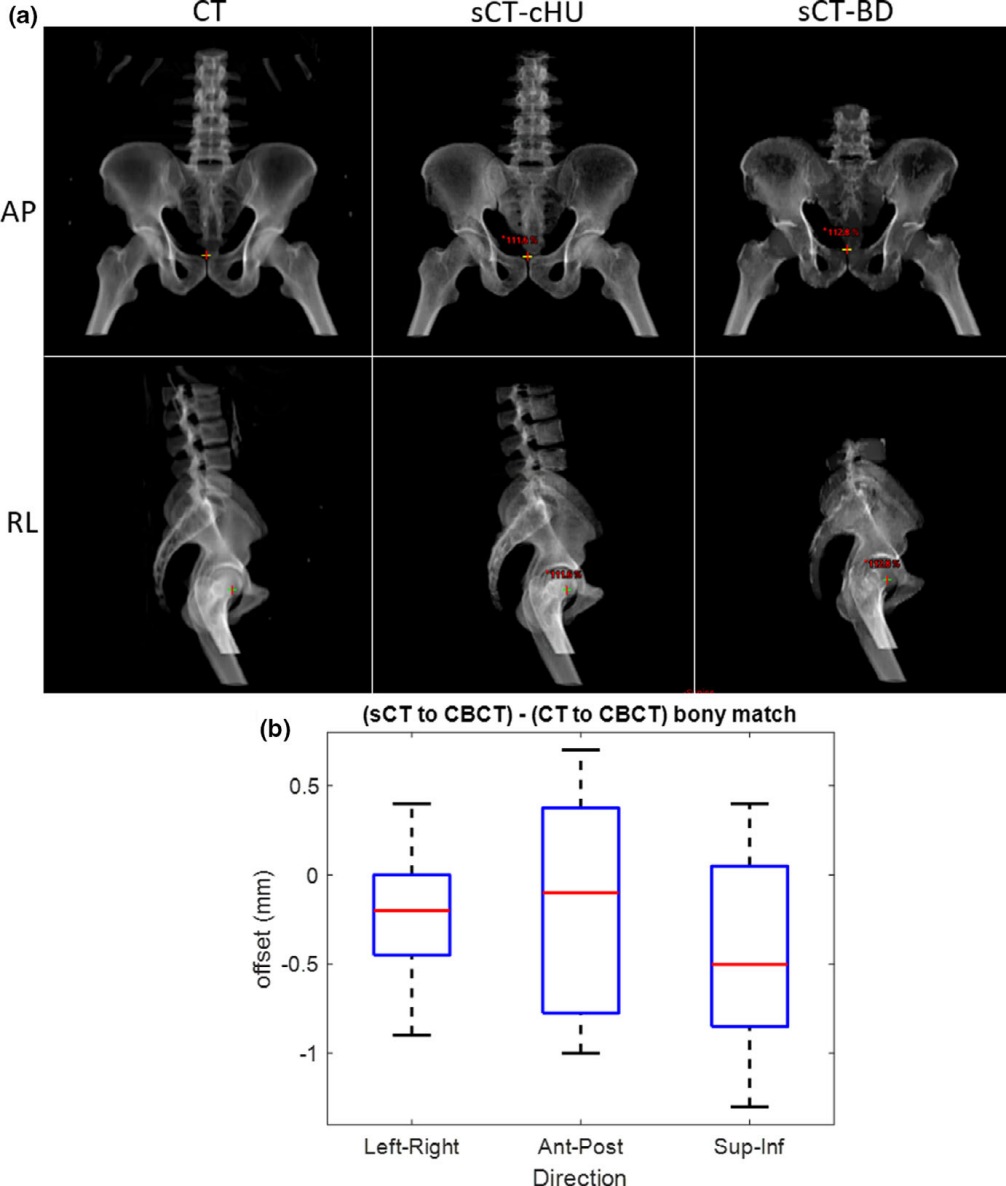
(a) Digitally reconstructed radiographs (DRRs) generated from CT (left) and continuous HU (cHU) sCT (middle), bulk density (BD) sCT (right) (b) Box plot of bony alignment differences between CBCT to CT and CBCT to sCT from 19 on‐treatment CBCTs from three patients. (CT: computed tomography; sCT: synthetic computed tomography; HU: Hounsfield unit; AP: anterior–posterior, RL: right–left, CBCT: cone beam computed tomography).

For the evaluated 12 cases with variety of treatment sites and setup positions, the MAE (average ± standard deviation) of bone, soft tissue, and total body between the sCT and CT are 120.9 ± 15.4 HU, 33.4 ± 4.1 HU, and 38.8 ± 4.0 HU, respectively. Detailed case‐by‐case results from all evaluated patients are shown in Table 2. The average PCC between sCT and CT‐derived DRR for both the AP and RL orthogonal projections were 0.97 and 0.98, respectively. DRRs generated from the planning CT and sCT of one example case can be seen in Fig. 6(a). Of the 19 CBCTs, the (average ± standard deviation) offset between CT and sCT as reference was (LR, AP, SI) = (0.19 ± 0.35, 0.14 ± 0.60, 0.44 ± 0.54) mm. A box plot of the resultant offset in all three directions is shown in Fig. 6(b).

**TABLE 2:** Tissue‐specific mean absolute error (MAE) between synthetic CT and CT for bone, soft tissue, and body (CT: computed tomography; HU: Hounsfield unit, Pt: patient).

Pt #	Treatment site	Tissue‐specific MAE (HU)		
		Bone	Soft tissue	Body
1	Gynecological	107.8	37.8	41.2
2	Gynecological	129.2	35.4	39.9
3	Gynecological prone	119.2	27.9	32.6
4	Gynecological periurethral	134.9	26.6	32.2
5	Rectum	90.9	38.2	42.9
6	Prostate bed	113.2	37.2	45.0
7	Prostate	135.8	31.3	40.5
8	Prostate	124.4	34.7	39.7
9	Prostate	139.5	32.3	39.0
10	Prostate and lymph nodes	108.2	32.1	36.4
11	Prostate and lymph nodes	138.7	28.9	34.6
12	Prostate and lymph nodes	109.0	38.1	41.4
Average		120.9	33.4	38.8

MAE of bone, soft tissue, and total body of the sCT‐BD approach compared with the sCT‐cHU approach is shown in Table 3. sCT‐cHU has lower MAE values than that of sCT‐BD for all compared tissue volumes as expected, particularly significant decrease of up to 40.3 HU was shown for the MAE in bone. A HU profile comparison of the two methods and the CT is also shown in Fig. 2 for an example case, where a closer agreement between the sCT‐cHU to CT can be seen.

**TABLE 3:** Mean absolute error (MAE) comparison for continuous HU synthetic CT (sCT‐cHU) and bulk density synthetic CT (sCT‐BD) to CT for two example cases (CT: computed tomography; HU: Hounsfield unit).

	MAE		
	Bone	Soft tissue	Body
Prostate			
sCT‐cHU	125.4	32.5	38.1
sCT‐BD	137.1	39.6	45.9
Prostate and lymph nodes			
sCT‐cHU	108.1	32.2	37.0
sCT‐BD	148.4	37.7	44.8

## 4 DISCUSSION

Successful clinical implementation of MRI‐only treatment planning have been reported from multiple institutions across the world but remains primarily in prostate cancer patients.^7^, ^8^, ^11^, ^12^ Wider utilization of MRI‐only treatment planning to maximize the benefit from the superior soft tissue contrast in MRI remains to be seen in other pelvis disease sites such as rectal and gynecological cancers. In rectal cancer, MRI is the most accurate tool for local cancer staging^13^, ^14^ and a powerful method to determine best course of treatment.^15^, ^16^, ^17^ In gynecological cancers, consensus remains that MRI is significantly more reliable than CT for the delineation of gross tumor volume, adjacent uterine tissue, and superior/inferior bladder extent.^18^, ^19^ Studies have also demonstrated that MRI‐assisted dose escalation in gynecological brachytherapy allowed for 10–20% overall survival gains while reducing urinary and gastrointestinal late morbidity.^20^, ^21^, ^22^

In a multi‐institutional study setting, we investigated the soft tissue visualization, dosimetric accuracy, and tissue‐specific HU similarity of a commercially available sCT‐cHU approach, MRCAT general pelvis. Our analysis suggest that this approach provided soft tissue and bone contrast on the sCT that is comparable to CT. Two‐stack acquisition enabled geometrically accurate MR as well as synthetic CT images and allowed anatomic coverage up to L1–L3 vertebrae to enable treatment of superior nodes including para‐aortic nodes. sCTs reconstructed successfully in both prone and supine positions as well as slight frog‐legged position used for gynecological setups. Soft tissue visualization scoring demonstrated high confidence in bladder, mesorectum and prostate rectum interfaces. All soft tissue visibility was substantially improved from the widely utilized prostate only sCT‐BD approach, as demonstrated in Fig. 2. In prostate cases, the rectal spacer showing as darker contrast compared with prostate and rectum allows for improved prostate and rectum boundary delineation when compared with CT. This has huge implication for CT‐cone‐beam computed tomography (CBCT) alignment on a linac where the spacer on the CT can only be displayed by the spacer contour. Even though fiducial visualization results were less consistent among the study cohort, it is a large improvement compared with the widely utilized bulk density approach in which fiducials are barely visible in the sCT.

In terms of dosimetry, the average percent difference between sCT‐cHU and CT were less than 1% for all evaluated PTV and OAR dose metrics across all disease sites. The rectal cancer cohort appear to have a more noticeable positive offset in overall distribution of the PTV D_95_, with a percent dose‐volume histogram (DVH) difference centered around 0.8%. This result suggests that the evaluated sCT‐cHU method overestimates PTV dose coverage by a small amount in rectal cancer cases. While this difference is clinically acceptable, it may be attributed to the fact that MRCAT general pelvis classifies air within the defined external body contour as soft tissue, in combination with the higher chance of air cavities within rectal cancer target volumes.

The average MAE of the entire body contour was 38.8 HU, consistent with the lower end of values reported in literature that ranges from 36.5 to 75.0 HU.^9^, ^23^, ^24^, ^25^ MAE analysis with whole body contour is less sensitive to HU changes due to the large ratio of soft tissue out of the whole body in contrast to bone. Therefore, tissue‐specific quantitative MAE analysis was performed to separate out and better understand the HU differences in bone. From available literature with tissue‐specific MAE values in the pelvic region,^24^ the tissue‐specific MAE values of was reported was 49.1 HU for intra‐pelvic soft tissues, 129.1 HU for bone marrow, and 274.4 HU for bony tissues. Our analyzed cohort of 12 cases shows average MAE of 120.9 HU for bone and 33.4 for soft tissue, indicating great HU agreement for the bony anatomy as well. Additional investigation directly comparing the tissue‐specific MAE to CT of the sCT‐BD approach and sCT‐cHU revealed that the latter provides an improvement of up to 40.0 HU in bone, and 7.8 HU in whole body contour. While Kemppainen et al. suggested there was not significant difference in dosimetric performance found between these two methods, the higher similarity in HU from the sCT‐cHU approach was shown to improve AP positioning precision due to improved visualization of the pubis.^9^

This study serves as a complement and extension to Kemppainen et al. (2019), where extensive evaluation of the same commercial sCT‐cHU platform from a single institution was first performed. The focus of their study was dosimetric and geometric evaluation of MRCAT general pelvis sCT. Their IGRT analysis showed mean DRR positioning accuracy within 0.3 mm in the AP, SI, and RL directions. For CBCT bone‐based positioning, the mean offset was also shown to be 0.1 mm. In terms of fiducial marker‐based IGRT workflows, the alignment relies on contours of the MRI‐localized fiducials rather than visualization of the fiducials themselves on the generated sCT. Fiducial based CBCT localization was extensively evaluated for the sCT‐BD approach,^7^ showing mean deviation of <1.0 mm. With a multi‐institutional perspective, comprehensive tissue‐specific MAE analysis that further separates out the overall performance of HU similarity, and the addition of quantitative tissue visibility scoring, this study provides additional confidence in the performance of this sCT‐cHU approach for clinical use.

## 5 CONCLUSIONS

MRCAT general pelvis with continuous HU generated realistic sCTs and DRRs to enable MRI‐only planning for general pelvis anatomy. Two‐stack acquisition enabled geometrically accurate MRI as well as sCT images and allowed anatomic coverage up to L1–L3 vertebrae to enable treatment to superior nodal volumes. The extension to general pelvis treatment sites allow for the benefit of MRI‐only planning to be realized in a larger patient population.

## CONFLICTS OF INTEREST

Memorial Sloan Kettering Cancer Center (MSKCC, New York, NY, USA), Turku University Hospital (TUH, Turku, Finland), and the Netherlands Cancer Institute (NKI, Amsterdam, The Netherlands) have signed a master research agreement with Philips Healthcare. Dr. Uulke Van der Heide receives research funding from Philips Healthcare.
